# Commentary: Contribution of Interoceptive Information to Emotional Processing: Evidence from Individuals with Spinal Cord Injury

**DOI:** 10.3389/fnint.2016.00040

**Published:** 2016-11-18

**Authors:** Giuliana Lucci, Patrizia Sablone, Erika Nista

**Affiliations:** ^1^Department of Psychology, University of Rome ‘La Sapienza’Rome, Italy; ^2^IRCCS Fondazione Santa LuciaRome, Italy

**Keywords:** spinal cord injuries, emotions, mood disorders, interoception, pain

We read with interest the study that has been recently published by Pistoia and colleagues on *Journal of Neurotrauma* (Pistoia et al., 2015). In their work, the authors addressed the longstanding issue concerning the influence of somatic inputs on emotional processing, comparing emotional processing of healthy and complete spinal cord injury (SCI) individuals. Since a complete spinal cord transection causes interruption of the neural flow from the periphery to the brain, SCI represents a useful model for testing the role of somatic inputs in emotional processing. The authors compared performance of healthy and complete SCI individuals in two emotional tasks, i.e., recognition of facial expressions and judgment of emotionally evocative scenes. In summary, Pistoia and co-workers observed difficulties in self-assessing emotionality toward fearful and angry scenes in SCI individuals, despite having normal performance in recognizing emotional faces. The authors explained their results invoking the simulationist theory of emotion recognition.

We have much appreciated this work, primarily because of its contribution in clarifying such a sensitive issue for people affected by SCI. We wish to take the opportunity we were given by reading this paper to highlight some key-points that in our opinion should be taken into account, when investigating how body–brain disconnection affects emotional processing.

For a start, it would be advisable to combine affective stimuli administration with physiological responses recording. As shown by literature on emotions in healthy samples, the subjective perceived stimuli valence and arousal are related to heart rate (Kreibig, 2010), skin conductance (Kreibig, 2010), facial electromyography (Dimberg et al., 2002), and event-related potentials (Hajcak et al., 2010). Neglecting physiological responses prevents from causally and univocally relating altered subjective feelings to body–brain disconnection.

Secondly, in investigating emotional processing in SCI, it must be taken into account the key role of interoception (i.e., the perception of internal bodily signals arising from the organs—e.g., heart, viscera, muscles) in emotional experience and cognition (Seth and Critchley, 2013), as partially done by Pistoia and colleagues. Spinal cord injury can indeed cause several physical sequelae that, by their very nature, could prevent from mapping internal bodily state and then affect emotional processing. We are referring to autonomic dysfunctions (for a review, see Hou and Rabchevsky, 2014), including cardiovascular (Partida et al., 2016) and gastrointestinal (Ebert, 2012) diseases, or autonomic dysreflexia. Autonomic dysreflexia (AD) is a sudden and uncontrolled sympathetic response triggered by stimuli below the level of injury. AD is characterized by elevation of arterial blood pressure, alteration of heart rate and feeling of anxiety (Krassioukov et al., 2009). In any case, the presence and the severity of autonomic dysfunctions could make the pattern of emotional response very different from case to case. Further, in their seminal work, Montoya and Schandry (1994), along with lower interoceptive accuracy and less intense emotionality in SCI than healthy individuals, observed a general positive correlation between interoceptive accuracy and emotional experience, endorsing the central role of interoception in modulating emotional experience.

These data together make advisable to assess interoception in all of its dimensions (Garfinkel et al., 2015) when investigating emotional processing in SCI individuals.

SCI patients frequently suffer from mood disorders, such as depression (Kreuter et al., 1998) and anxiety (Kennedy and Rogers, 2000; Craig et al., 2009) that, if not treated, can persist for two years or more after their onset. A relatively large literature on emotions documented that mood disorders go with emotion processing alterations (Leppänen, 2006; Elliott et al., 2011). Furthermore, psychological diseases get even worse when neuropathic pain, frequently affecting SCI individuals (Finnerup and Jensen, 2004), is present: comorbidity of mood disorders and neuropathic pain is observed in about one-third of SCI individuals (Gustorff et al., 2008). With regard to pain, it is noteworthy its close link with interoception (Tracey and Mantyh, 2007). Therefore, since the complex scenario mutually involving mood, pain, and interoception in emotionality, it would be helpful assessing the presence and the gravity of both mood disorder and neuropathic pain in investigating emotions in SCI individuals.

Finally, to complete the picture, we must not forget that spinal cord injuries radically affect the life of those suffering from them, causing several losses (i.e., physical, psychological, relational, existential) with whom SCI individuals have to cope. The trauma itself and the coping with the new special life could change subjective emotional framework. Then, to get around this potential confounding variable a control patient group, e.g., patients having breast or prostate removed, could be included when investigating emotions after body–brain disconnection.

We should like to conclude by remarking emotional processing as key issue in the recovery and rehabilitation process of clinical populations, and therefore also for individuals with spinal cord injury, then further works on emotional processing in SCI individuals are necessary and desirable in order to keep trying to improve the quality of their life.

## Author contributions

All authors participated in the preparation and discussion of the commentary.

### Conflict of interest statement

The authors declare that the research was conducted in the absence of any commercial or financial relationships that could be construed as a potential conflict of interest.
